# All-Weather Thermal Simulation Methods for Concrete Maglev Bridge Based on Structural and Meteorological Monitoring Data

**DOI:** 10.3390/s21175789

**Published:** 2021-08-28

**Authors:** Ao Wang, Zongkai Zhang, Xiaoming Lei, Ye Xia, Limin Sun

**Affiliations:** 1 Department of Bridge Engineering, Tongji University, Shanghai 200092, China; tjwangao@tongji.edu.cn (A.W.); lmsun@tongji.edu.cn (L.S.); 2 China Overseas Construction Limited, Shenzhen 518005, China; zjzhangzongkai@cohl.com; 3 State Key Laboratory of Disaster Reduction in Civil Engineering, Tongji University, Shanghai 200092, China; 4 Shanghai Qi-Zhi Research Institute, Shanghai 200092, China

**Keywords:** concrete bridge structure, temperature field, solar radiation, meteorological monitoring, thermal boundary, all-weather FE method, experimental verification

## Abstract

Thermal energy exchange induces non-uniform temperature distribution on the concrete bridge structures, leading to variation of static and dynamic properties of structural systems. The finite element method can facilitate thermal simulation and predict the structural temperature distribution based on heat flow theories. Previous studies mainly focused on the daytime with sunny weather, and the effects of solar shadow distribution were not fully considered or even ignored. In this paper, a systematic all-weather thermal simulation method was proposed to investigate the temperature distributions of concrete maglev bridges. The solar shadow distribution on the bridge surface could be accurately simulated to determine the solar radiation-imposed range. A meteorological station and some thermocouples were installed on a real concrete maglev bridge to obtain the real-time structural temperatures and environmental conditions. Its temperature distribution is also simulated using the proposed method within the 27 monitoring days in Summer. Results show that the simulated structural temperature matches well with the measured results under various weather conditions, except that of the east structural surface. Moreover, the simulation method acquired a higher accuracy under overcast or rainy weather due to weaker solar radiation effects. Both the numerical results and experimental records illustrated that direct solar radiation dominates the thermal energy exchange under sunny or cloudy conditions. The proposed methodology for temperature field simulation is oriented by all-weather prediction of structural temperature, which is reliable for concrete bridge structures with the help of accurate measurement of real-time solar radiation.

## 1. Introduction

Concrete bridges are generally constructed in open areas and exposed to the environment, and the structural temperature fields are heavily affected by the energy exchanged between bridge structures and surrounding environments [1,2]. For concrete bridge structures with low conductivity of concrete material, which are sensitive to thermal loads, the uneven distribution and fluctuation of structural temperature cause undesirable deformations and stresses [3,4], leading to variation of the entire structure’s static and dynamic properties [5,6]. Many studies on temperature fields were conducted by structural health monitoring techniques. A great number of structural health monitoring (SHM) systems with temperature monitoring functions have been installed on some constructed bridges in the world [7,8,9], which provides an effective approach to investigate the temperature field of real bridge structures based on monitored data [10].

Scaled models and partial girder segments were mostly adopted in previous studies to analyze the impact of environmental thermal loads on temperature distribution, because the enormous sensors required on large structures are often limited due to the high cost of instrumentation. Liu et al. [11] set up a numerical simulation and experimental study on an H-shaped steel specimen considering the shadow of solar radiation based on 3-day temperature measurements in summer. The web depth and flanges width of the specimen was 200 mm, whereas the length was 500 m. Although the small specimen obtained better temperature field simulations due to the simple structures, the actual systems were more complex for numerical simulation. Abid et al. [12] established a full-scale concrete box-girder segment with 62 thermocouples and a weather station to analyze distributions of temperature based on continued 1-year data acquisition. Empirical formulas were proposed to predict the vertical and lateral temperature gradients based on meteorological data. Later the same approach was used to study the temperature distributions in a small concrete-encased steel specimen in addition to finite element investigation [13]. A large experimental model of a box-girder arch was established by Wang et al. [14] to evaluate the effect of nonlinear temperature gradients on arch structure; meanwhile, the finite element simulation was verified by 1-day experimental measurements data. The model arch span was 59.33 m, and the arch rise is 13.41 m. The solar radiation effect was not explicitly considered in this study. Xia et al. [15] carried out an experiment and numerical analysis on a simply supported concrete slab specimen (3.0 m span, 0.8 m wide, and 0.12 m thickness) to investigate the temperature-induced variation of structural frequencies. For the thin slab without solar radiation, the structural temperature simulation fit measured records well.

With the rapid development of numerical simulation, the finite element (FE) method can facilitate thermal numerical simulation using heat flow equations [16,17]. Therefore, the structural temperature fields can be simulated considering different thermal boundary conditions. Several environmental factors, including solar radiation, weather conditions, bridge orientation, material, cross section form, etc., determine the thermal boundary conditions [18]. Among these factors, solar radiation has a significant effect on the accuracy of numerical simulation [19]. The temperature field simulation accuracy is usually affected by the real-time simulation of solar shadow and applying a suitable solar radiation flux model. In the literature mentioned above, the simulation algorithm of solar shadow was conducted by the experimental analysis of a simple H-shaped steel specimen [11], which was not applicable for the real bridge with complex structural shapes. Therefore, an algorithm for simulation of the solar shadow is first required for numerical analysis of the structural temperature field. In terms of day timescale, a complete simulation method based on continuous meteorological monitoring to predict the nighttime temperature distribution of the structure.

In the previous researches, three common solar radiation flux models were applied in boundary conditions [20]. The American Society of Heating, Refrigerating and Air-Conditioning Engineers (ASHRAE) clear-sky model [21] was a widely used empirical formula obtained by the measured meteorological data of solar radiation. The direct solar radiation and scattered radiation were calculated based on three empirical coefficients. Hottel model [22] was used to calculate the direct solar transmittance and scattered transmittance through empirical formulas to determine the components of solar radiation. The key point of Power-law model [23] was to determine the atmospheric transparency coefficient by the empirical formula. In the above-mentioned theoretical solar radiation models, the empirical coefficients relied on a great number of measured data and statistical results in the past. However, the three mentioned solar radiation models were only applicable to calculate solar radiation in sunny weather conditions rather than cloudy or rainy ones, which means the calculation accuracy will reduce for not considering the change of solar radiation caused by cloud shielding during a day. Besides, the empirical atmospheric transparency coefficient of the Power-law model obtained in the past year may not be suitable due to atmospheric conditions changes caused by human activities in recent years. As a result, a solar radiation flux calculation method suitable for various weather conditions can realize the accurate all-weather simulation of the temperature field.

Focusing on thermal effects on structural temperature distribution, this paper carried out an all-weather numerical simulation methodology of the structural temperature field, based on the real-time meteorological monitoring data of the bridge site and a simulation algorithm of real-time solar shadow, to predict the structural temperature distribution of concrete bridges. The logic of the paper is as follows: (i) the experimental setup of a maglev bridge in service is introduced, in which thermocouples were instrumented on the structural surfaces, and a meteorological station was established at the bridge site; (ii) the theory of thermal boundary conditions for the three-dimensional transient heat analysis is elaborated with a simulation method of solar shadow; (iii) overall framework of all-weather thermal numerical simulation is presented and applied to extensive analysis of the experimental structure, in which initial temperature values and equivalent boundary conditions were adopted; and (iv) the numerical results and measured records of structural temperature are presented and discussed to demonstrate the proposed methodology, especially for considering the weather conditions during 27 days. The evaluation of the methodology and further study work is summarized in conclusions.

## 2. Theory of Heat Conduction

### 2.1. Thermal Boundary Conditions

The three-dimensional transient heat conduction differential equation written as Equation (1) was employed to analyze the temperature field of the concrete bridge structure [18,24,25]. It was assumed that the structural system has no internal heat source, and it is presented with uniform mass and isotropy of material properties.

(1) $$\frac{\partial }{\partial x}\left(\lambda \frac{\partial T}{\partial x}\right)+\frac{\partial }{\partial y}\left(\lambda \frac{\partial T}{\partial y}\right)+\frac{\partial }{\partial z}\left(\lambda \frac{\partial T}{\partial z}\right)=\rho c\frac{\partial T}{\partial t}$$

where ρ, c, and λ represent the density ($\mathrm{kg}\cdot m^{-3}$), specific heat capacity ($J\cdot {\mathrm{kg}}^{-1}\cdot K^{-1}$), and thermal conductivity ($W\cdot m^{-1}\cdot K^{-1}$) of concrete material, respectively; T is the temperature of a point on the structural surface at a certain time t.

The initial condition for the equation solution is the temperature distribution at the initial time [26], which can be written as Equation (2):

(2) $${T\left(x,y,z,t\right)|}_{t=0}=T_{0}\left(x,y,z\right)$$

### 2.2. Thermal Boundary Conditions

**Figure 1:**
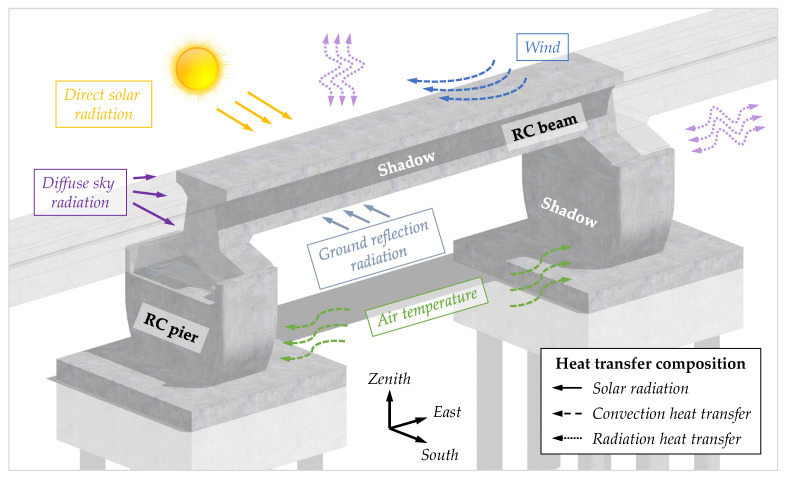
Heat flow schematic diagram of the maglev bridge.

For a concrete bridge structure exposed to the environment, the heat transfer acting on the structural surface includes solar radiation, convection heat transfer, radiation transfer, as shown in Figure 1. Therefore, the thermal boundary condition of the structural surface at a certain time is defined as Equation (3):

(3) $$\lambda {\frac{\partial T}{\partial n}|}_{\Gamma }=q_{s}+q_{c}+q_{r}$$

where $q_{s}$, $q_{c}$, and $q_{r}$ represent the shortwave solar radiation flux ($W\cdot m^{-2}$), heat convection flux ($W\cdot m^{-2}$), and radiation heat transfer flux ($W\cdot m^{-2}$), respectively; Γ is the boundary surface of the structure object; n is the external normal line direction of the boundary surface.

### 2.3. Solar Radiation Flux

#### 2.3.1. Geometric Parameters

**Figure 2:**
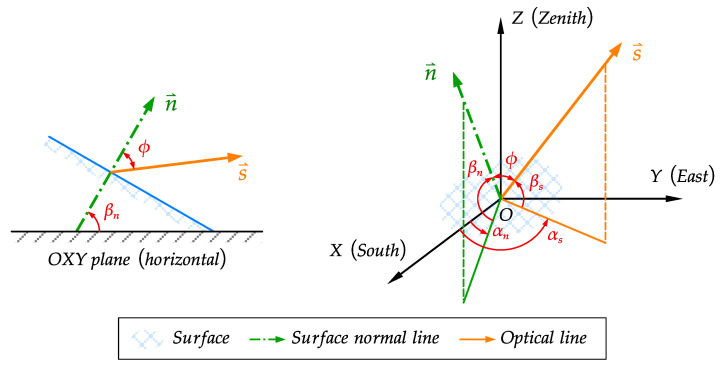
Diagram of coordinate system and vectors.

For each structural surface under solar radiation, a spatial coordinate system OXYZ is established to define each relevant angle, as shown in Figure 2. The origin point O is located to the illuminated surface, OXY plane is parallel to the horizontal plane, the X-axis points to the geographical south direction, the Y-axis points the geographical east direction, and the X-axis points to the zenith and is perpendicular to the horizontal plane. The relevant angles are defined as follows.

$\alpha_{n}$: azimuth of the surface, which is obtained by the geometric shape of each structural surface.

$\beta_{n}$: the inclination of the surface, which is the angle between the external normal line of the structural surface and the OXY plane, obtained by the geometric shape of each structural surface, and $\left|\beta_{n}\right|\leq 90{}^{\circ}$; besides, the structural surface is not exposed to the sun radiation when $\beta_{n}\leq 0{}^{\circ}$.

$\alpha_{s}$: solar azimuth, and $0{}^{\circ}<\alpha_{s}\leq 90{}^{\circ}$ for southeast direction, $-90{}^{\circ}\leq \alpha_{s}<0{}^{\circ}$ for southwest direction.

$\beta_{s}$: solar altitude angle, which is the angle between the optical line and the OXY plane.

ϕ: solar incident angle, which is the angle between the external normal line of the structural surface and the optical line, and 0°≤ϕ≤180°; besides, the structural surface is not exposed to the sun radiation when 90°≤ϕ; the relation between ϕ and other angles can be derived as Equation (4):

(4) $$\begin{array}{ll} \cos\phi = & \left(\sin\varphi \cos\beta_{n}-\cos\varphi \cos\alpha_{n}\cos\beta_{n}\right)\sin\delta \\ & +\left(\cos\varphi \sin\beta_{n}+\sin\varphi \cos\alpha_{n}\cos\beta_{n}\right)\cos\delta \cos\tau \\ & +\sin\alpha_{n}\cos\beta_{n}\cos\delta \sin\tau \end{array}$$

where φ is the latitude of each structural surface, which is taken as the latitude of the bridge site approximately, and 0°<φ≤90° for the northern hemisphere; δ: daily solar declination is calculated by empirical formula [27] as Equation (5), in which N is the day order number of the year;

(5) $$\delta =23.45^{{}^{\circ}}\sin\left[\frac{360{}^{\circ}}{365}\left(284+N\right)\right]$$

τ: solar hourly angle, and τ=(12−t)×15°, in which t is the real solar time (h) determined by the longitude (α) of the bridge site as Equation (6):

(6) $$t=BST-\frac{\left(120{}^{\circ}-\alpha \right)}{15{}^{\circ}}+t_{d}$$

where BST is Beijing standard time (h); $t_{d}$ is time difference (h) calculated by empirical formula [28] as Equation (7), in which $\theta_{n}=360{}^{\circ}\left(N-81\right)/364$.

(7) $$t_{d}=0.165\sin2\theta_{n}-0.025\sin\theta_{n}-0.126\cos\theta_{n}$$

Further, the relation between $\beta_{s}$ and other angles can be derived as Equation (8):

(8) $$\sin\beta_{s}=\cos\varphi \cos\delta \cos\tau +\sin\varphi \sin\delta$$

Finally, the relation between $\alpha_{s}$ and other angles can be derived as Equation (9):

(9) $$\cos\alpha_{s}=\frac{\sin\varphi \cos\delta \cos\tau -\cos\delta \sin\tau }{\cos\beta_{s}}$$

#### 2.3.2. Solar Radiation Calculation

For concrete structures exposed to solar radiation, the shortwave solar radiation flux ($W\cdot m^{-2}$) absorbed by the structural surfaces can be expressed as Equation (10):

(10) $$q_{s}=A_{s}q_{\phi }$$

where $A_{s}$ is the shortwave radiation absorption rate of the structural surface. The concrete value is generally between 0.55 and 0.70, and the lighter the surface color, the greater the value [29]. $q_{\phi }$: total solar shortwave radiation ($W\cdot m^{-2}$) projected onto the structural surface with arbitrary inclination, which consists of real direct solar radiation $I_{D\phi }$, diffuse sky radiation $I_{d\beta }$ and ground reflection radiation $I_{r\beta }$, and is illustrated as Equation (11):

(11) $$q_{\phi }=I_{D\phi }+I_{d\beta }+I_{r\beta }=I_{D}\cos\phi +\frac{1+\sin\beta_{n}}{2}I_{dH}+\frac{1-\sin\beta_{n}}{2}r_{e}I_{G}$$

where $I_{D}$ is the direct solar radiation flux ($W\cdot m^{-2}$) on the horizontal plane calculated by Equation (15), when the structural surface is not exposed to solar radiation, $I_{D}$ is equal to 0; $I_{dH}$: diffuse sky radiation ($W\cdot m^{-2}$) on the horizontal plane; $I_{G}$: total solar radiation flux ($W\cdot m^{-2}$) on the ground, which is measured by the radiometer at the bridge site; $r_{e}$: surface shortwave reflectance on the ground, and the value is 0.20 in most cases.

Based on the real-time measured total solar radiation flux $I_{G}$ records at the bridge site, the diffuse sky radiation $I_{dH}$ can be obtained by the empirical formula [30] as Equation (12):

(12) $$\frac{I_{dH}}{I_{G}}=\{\begin{array}{ll} 1.0-0.248k_{T} & k_{T}<0.35,\\ 1.557-1.84k_{T} & 0.35\leq k_{T}<0.75\\ 0.177 & 0.75\leq k_{T} \end{array},$$

where $k_{T}$ is atmospheric cleanliness index [30], and defined as Equation (13):

(13) $$k_{T}=\frac{I_{G}}{I_{0}}$$

where $I_{0}$ is daily solar constant ($W\cdot m^{-2}$) calculated by the empirical formula [31] as Equation (14):

(14) $$I_{0}=1367\left[1+0.033\cos\left(\frac{360{}^{\circ}N}{365}\right)\right]$$

Finally, direct solar radiation $I_{D}$ can be calculated by Equation (15):

(15) $$I_{D}=\frac{I_{bH}}{\sin\beta_{s}}=\frac{I_{G}-I_{dH}}{\sin\beta_{s}}$$

where $I_{bH}$ is the horizontal component of direct solar radiation flux ($W\cdot m^{-2}$).

### 2.4. Convection Heat Transfer Flux

Radiation convection heat transfer flux $q_{c}$ ($W\cdot m^{-2}$) received by the arbitrary structural surface is calculated by Equation (16).

(16) $$q_{c}=h_{c}\left(T_{a}-T_{\Gamma }\right)$$

where $h_{c}$ is coefficient of convection heat transfer ($W\cdot m^{-2}\cdot K^{-1}$) obtained by Equation (17), in which the measured records of air temperature $T_{a}$ (℃) and wind speed v ($m\cdot s^{-1}$) are adopted [32]; $T_{\Gamma }$: the structural surface temperature obtained by initial temperature value.

(17) $$h_{c}=2.6\times \sqrt[{4}]{\left|T_{a}-T_{\Gamma }\right|}+4.0v\;\left(v\leq 5.0\right)$$

### 2.5. Radiation Heat Transfer Flux

Radiation heat transfer flux $q_{r}$ ($W\cdot m^{-2}$) received by the arbitrary structural surface is calculated by Equation (18):

(18) $$q_{r}=h_{r}\left(T_{a}-T_{\Gamma }\right)-q_{ra}$$

where $h_{r}$ is the coefficient of radiation heat transfer ($W\cdot m^{-2}\cdot K^{-1}$) calculated by Equation (19):

(19) $$h_{r}=\varepsilon C_{0}\left(546+T_{a}+T_{\Gamma }\right)\left[{\left(273+T_{a}\right)}^{2}+{\left(273+T_{\Gamma }\right)}^{2}\right]$$

$q_{ra}$: oblique sky radiation ($W\cdot m^{-2}$) effect obtained by Equation (20):

(20) $$q_{ra}=\frac{1+\sin\beta_{n}}{2}\left(1-\varepsilon_{a}\right)\varepsilon C_{0}{\left(273+T_{a}\right)}^{4}$$

where $C_{0}$ is the Stefan–Boltzmann constant ($W\cdot m^{-2}\cdot K^{-4}$), and the value is defined as $5.67\times 10^{-8}$; ε: emissivity of the structural surface, and the value of concrete is 0.90 approximately; $\varepsilon_{a}$: coefficient of atmospheric radiation, and $\varepsilon_{a}=1-0.261\exp\left(-7.776\times 10^{-4}T_{a}^{2}\right)$, the value takes a constant of 0.82 approximately [29].

## 3. Thermal Simulation Methods

### 3.1. Simulation of Solar Shadow

**Figure 3:**
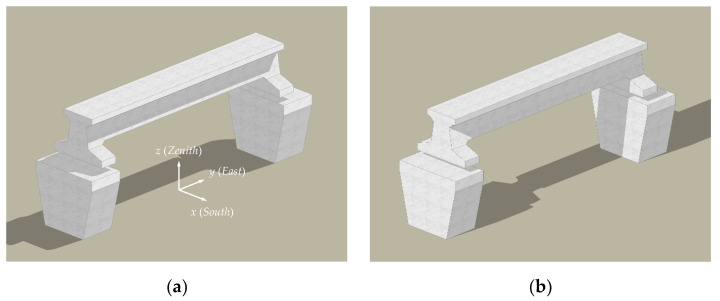
Simulation of solar shadow distribution on the structure on 23 July 2019. (**a**) 09:00; (**b**) 15:00.

The shadow distribution of solar radiation on the maglev bridge at 9:00 and 15:00 on 23 July 2019 was shown in Figure 3, rendered by a 3D modeling computer program. The results indicate that the solar shadow distribution on the bridge surface changes as the solar irradiation angle changes. Therefore, it is necessary to establish an effective solar shadow simulation algorithm to determine the solar radiation-imposed range accurately.

In this study, a method based on computer graphics [33] and the FE geometry model was proposed to simulate the distribution of solar shadow in numerical analysis. The cross-product method, the real-time solar azimuth $\alpha_{s}$, and solar altitude angle incident angle $\beta_{s}$ were applied to determine whether the intersection point of the optical line was located within the surface boundary. The procedure of the cross-product method is as follows.

**Figure 4:**
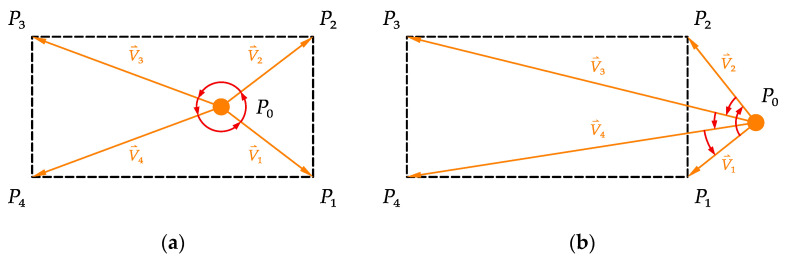
Schematic diagram of the cross-product method. (**a**) The point $P_{0}$ is on the plane $P_{1}P_{2}P_{3}P_{4}$; (**b**) the point $P_{0}$ is out of the plane $P_{1}P_{2}P_{3}P_{4}$.

Step 1: Select an element located on the surface of the FE model and obtain the center point coordinates of the external surface of the FE element.
Step 2: Generate an optical line from the center point corresponding to the real-time solar azimuth $\alpha_{s}$ and solar altitude angle incident angle $\beta_{s}$.
Step 3: Assume the optical line intersected with the infinite plane, on which the upper structural surface was located, at point $P_{0}$, and each corner point of the upper structural surface plane was $P_{1}$,$\;P_{2}$,$\;P_{3}$, and $P_{4}$ in order; then to get the vectors ${\vec{V}}_{i}=P_{i}-P_{0}$, i=1,2,3,4, and assume ${\vec{V}}_{5}={\vec{V}}_{1}$.
Step 4: If the directions of ${\vec{V}}_{i}\times {\vec{V}}_{i+1}$ (i=1,2,3,4) are all the same, the intersection point $P_{0}$ locates on the upper surface plane $P_{1}P_{2}P_{3}P_{4}$, that means the element is shaded, as shown in Figure 4a; otherwise, if the directions of ${\vec{V}}_{i}\times {\vec{V}}_{i+1}$ (i=1,2,3,4) are not the same, the intersection point $P_{0}$ locates out of the upper surface plane $P_{1}P_{2}P_{3}P_{4}$, that means the element is unshaded, as shown in Figure 4b.
Step 5: The element of the other surface in shadow induced by the upper structural surface plane can be determined by Step 1 to Step 4.

**Figure 5:**
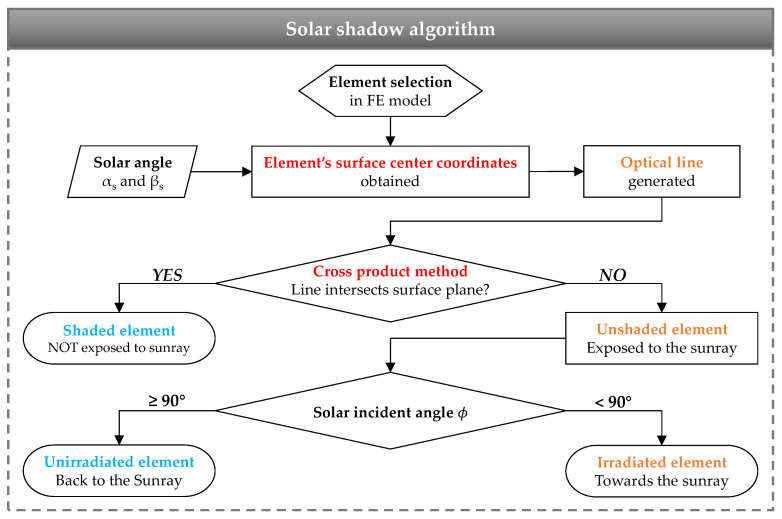
Flowchart of solar shadow simulation algorithm in FE model.

Furthermore, with extracting the external normal vectors of the elements, the real-time solar incident angle ϕ was calculated to determine whether the element is toward to sunray. Finally, the whole procedure of the simulation method of the solar shadow is summarized in Figure 5.

### 3.2. Thermal Simulation Methods Considering Solar Radiation Effects

**Figure 6:**
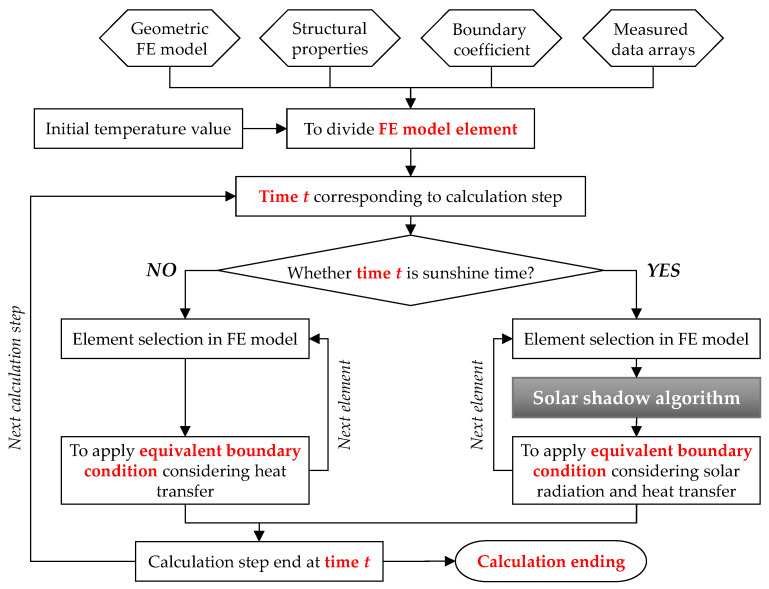
Framework of the numerical simulation considering solar radiation effects.

The main steps of numerical simulation on temperature distribution considering solar radiation effects in ANSYS software are summarized in Figure 6. The whole procedure is illustrated in detail below.

Stage 1: Establish a geometric FE model using the SOLID70 element with the appropriate element size according to the design drawing; to import the main structural parameters, including material properties and geography information as listed in Table 1.

Stage 2: Import the meteorological records during the whole experimental period as measured data arrays, consisting of the air temperature, wind speed, and total solar radiation on the ground; to import the initial temperature value.

Stage 3: Start the calculation step at the corresponding time and judge whether there is sunray at the corresponding computing time.

Stage 4: If the value of solar radiation at the corresponding time is 0, to apply the equivalent boundary condition considering convection heat transfer and radiation heat transfer flux calculated according to Section 2.3 and Section 2.4, and then carry out a transient thermal calculation.

Stage 5: If the value of solar radiation at the corresponding time is not 0, apply the solar shadow algorithm and judge whether elements are exposed to direct solar radiation according to Section 3.1; apply the equivalent boundary condition considering solar radiation, convection heat transfer and radiation heat transfer flux calculated according to Section 2.2, Section 2.3 and Section 2.4, and then carry out a transient thermal calculation.

Stage 6: The calculation step at the corresponding time finishes; start the next calculation step from Step 3 to Step 5 until the calculation ends.

Stage 7: Obtain the numerical simulation results from the FE model to analyze the temperature distribution on the concrete structural members.

## 4. Experimental Program

### 4.1. Description of a Concrete Maglev Bridge

**Figure 7:**
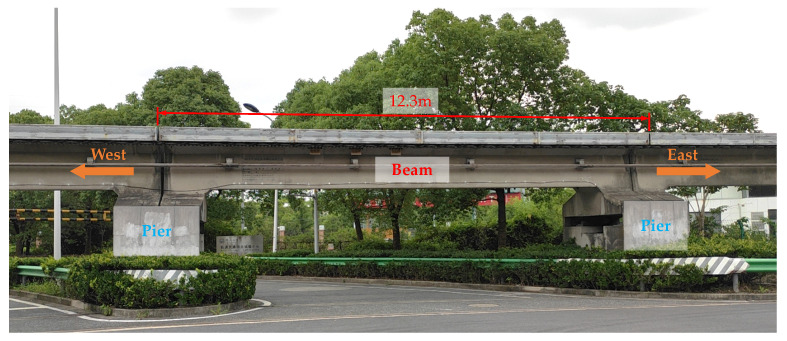
The concrete maglev bridge.

**Figure 8:**
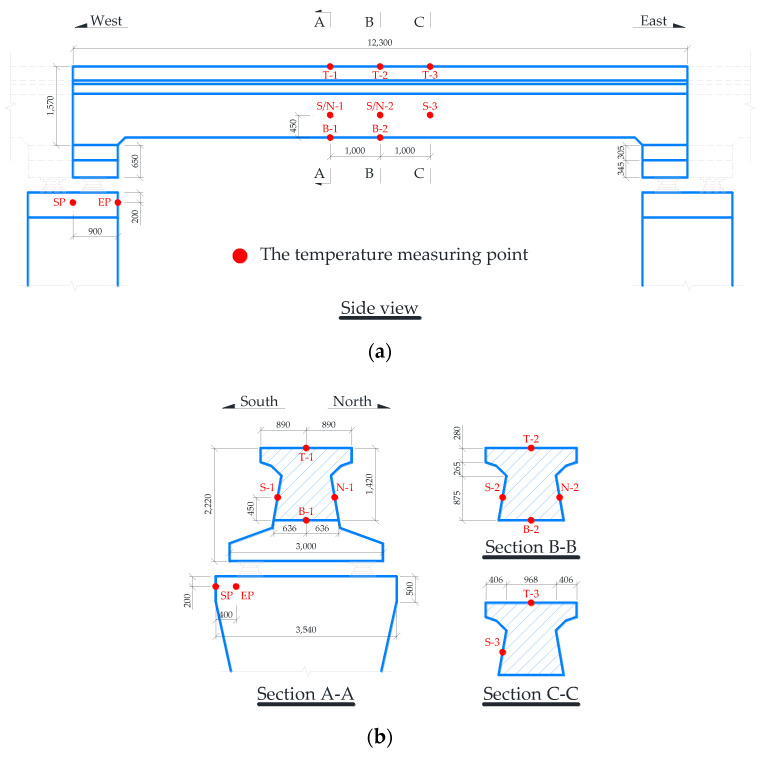
Arrangement of temperature measuring points (unit: mm). (**a**) Elevation view; (**b**) key section layout.

In this study, a concrete maglev bridge, with a simply supported straddle-type monorail track beam and two piers, was tested to obtain the structural surface temperature under regional meteorological effects in summer, as shown in Figure 7. The FE model of the concrete maglev bridge was also created with ANSYS software to simulate the temperature distribution based on meteorological monitoring records. The length of the simply supported beam is 12.3 m. The beam adopted a T-shaped solid section to meet the Maglev track arrangement, as shown in Figure 8.

**Figure 9:**
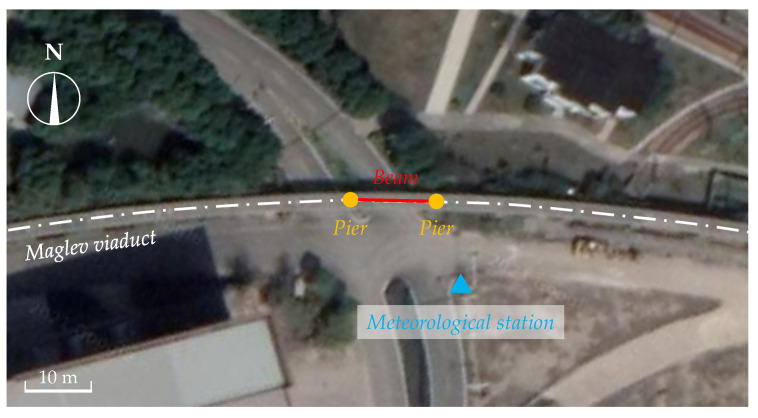
Arrangement of the experimental site.

As shown in Figure 9, the beam is located at a flat and open site and thus will not be shaded from solar radiation by surrounding buildings or trees, which excluded the shadow of solar radiation from the surrounding environment. Moreover, the longitudinal axis of the beam was in an east-west direction with an azimuth angle of $\pm 90^{{}^{\circ}}$ (the azimuth angle of the south is equal to $0^{{}^{\circ}}$).

### 4.2. Experimental Instrumentation Setups

#### 4.2.1. Measurement of Structural Surface Temperature

For the constructed concrete maglev bridge in use, thermocouples (Pt100) were installed on the exterior structural surface to measure the concrete temperature for no obvious damage to the structure. The thermocouples were embedded within bored holes with approximately 1.5 to 2.0 cm deep and filled with cement mortar, which has a similar thermal conductivity with surrounding concrete. The accuracy of the adopted thermocouple is ±0.15 ℃ and with the measurement range of −50 to 250 ℃. The thermocouples were distributed in six groups according to their surface orientation. The groups were the top surface (TS), bottom surface (BS), south surface (SS), and north surface (NS) of the beam, and south surface (SP) and east surface (EP) of the west pier, which consists of 3 (T-1 to T-3), 2 (B-1 to B-2), 3 (S-1 to S-3), 2 (N-1 to N-2), 1 (SP), and 1 (EP) thermocouples, respectively. All temperature measuring points were arranged as Figure 8. Meanwhile, the 16-channel electric data collector for structural temperature measuring was set up and powered by serially connected batteries during experimental period. The experimental data were recorded at a sampling rate of 1 time per ten minutes constantly from July 2019 to August 2019. Each group of adjacent sensors was regarded as a measuring point on the corresponding structural surface, and the structural temperature of the measuring point was obtained by the average value of the sensors records.

#### 4.2.2. Meteorological Monitoring Station

**Figure 10:**
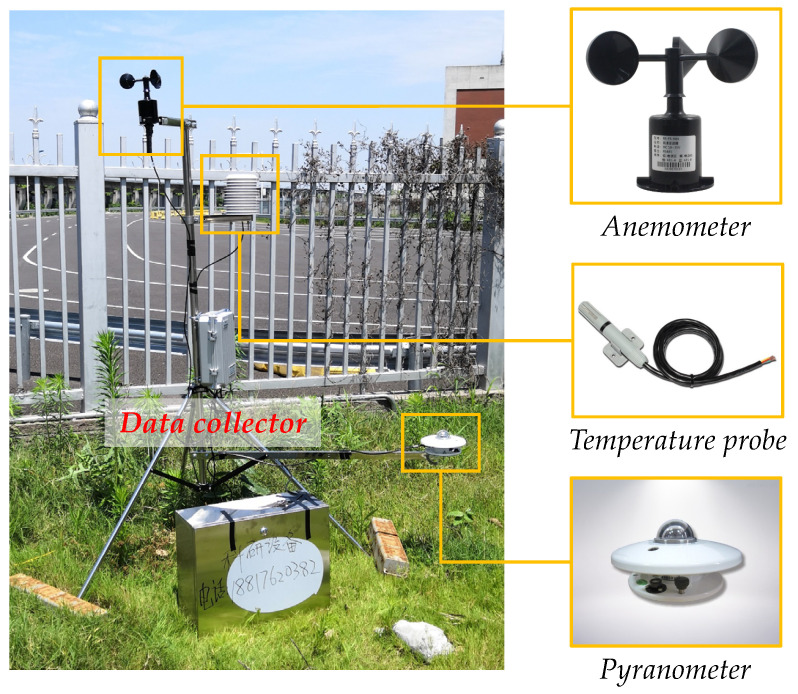
Composition of the meteorological station.

Air temperature, wind speed, and solar radiation are the main factors affecting the temperature distribution of the concrete beam. The meteorological station (BLJW-4), consisting of three sensors provided by Beijing Bolen-Jingwei Company, Beijing, China, was located at an open site ~10 m away from the south side of the experimental bridge to monitor the meteorological conditions, as shown in Figure 9. As shown in Figure 10, the temperature probe (BL-WS) was used to monitor the shade temperature of the air, the three-cup anemometer (BL-FX) was used to monitor the speed of the wind, and the pyranometer (BL-ZFS) was used to monitor the solar radiation intensity. The data collector (BLJW-4) obtained air temperature, wind speed, and solar radiation at a constant sampling rate of 1 time per ten minutes from July 2019 to August 2019, consistent with the structural temperature sampling rate.

## 5. FE Model Setups

### 5.1. Basic Information and Parameters

**Figure 11:**
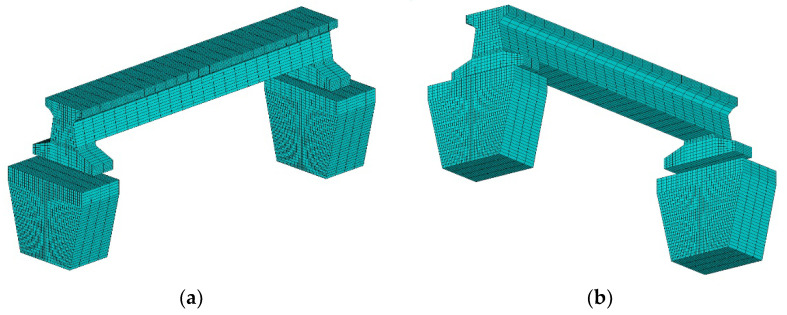
The three-dimension FE model with meshed elements. (**a**) Top view; (**b**) bottom view.

A numerical simulation method was adopted based on a transient FE temperature field simulation. The maglev bridge FE model with three-dimensional thermal element SOLID 70 was established in ANSYS 2020 R2 software. The mechanical boundary condition was not considered in the FE model for thermal analysis only, including the elastic connection of the beam and piers to simulate the supports and the fixed constraint at the bottom of the piers. The FE model has 19,152 elements and 21,892 nodes, as shown in Figure 11. As the temperature distribution on the mid-span cross section of the beam structure is the main concern for bridge structure, the small enough element mesh size on the cross section of the FE model is required to realize the higher solution accuracy. The concrete material [34] and geography parameters adopted in the FE model are listed in Table 1.

Based on the color comparison of each structural surface of concrete, the shortwave radiation absorption rate of each structural surface $A_{s}$ was determined. The value of top, bottom, south, and north surface on the beam is 0.95, 0.80, 0.65, and 0.70, respectively; the value of the south and east surface on piers is 0.65 and 0.90, respectively; and the value of rest surfaces on the maglev bridge is 0.70.

### 5.2. Thermal Boundary Conditions

#### 5.2.1. Initial Temperature Values

For the transient calculation of the structural temperature field, the initial condition is required to determine the convective heat transfer coefficient $h_{c}$ and radiant heat transfer coefficient $h_{r}$.

**Figure 12:**
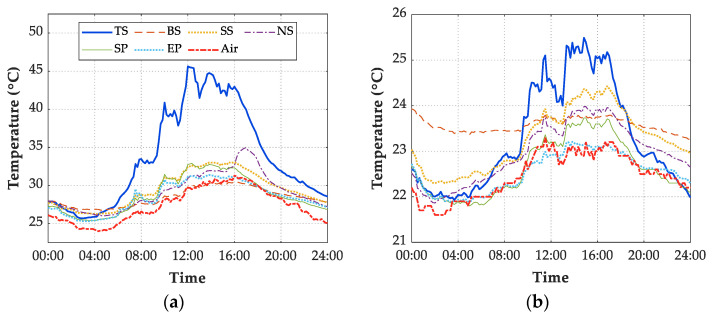
Temperature history of 6 measuring points on the bridge and air. (**a**) 07 July 2019; (**b**) 13 July 2019.

Figure 12 shows the temperature history of 6 measuring points on the structure and air on 7 July 2019 and 13 July 2019. The structural and air temperatures met minimum values before sunrise on 7 July 2019 with cloudy conditions. The minimum values of measuring points were relatively close to each other at nearly the same time. Such phenomenon of temperature history also appeared on 13 July 2019 with the rainy condition. Because the solar radiation effects on the surface temperature and air have gradually disappeared during the night, the structural temperature was uniform before sunrise. Overall, the average temperature value of the six measuring points can be obtained as the structural temperature before sunrise.

**Figure 13:**
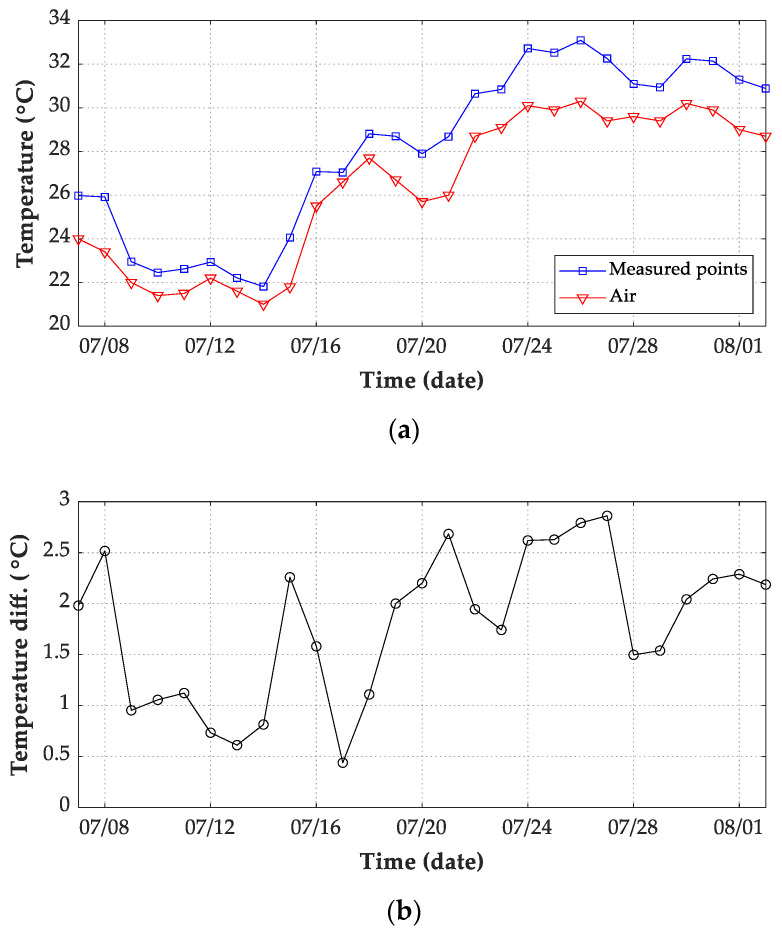
Daily air temperature records from 7 July 2019 to 2 August 2019. (**a**) Comparison between the average temperature of 6 measuring points with air temperature; (**b**) temperature difference (measured structural temperature minus air temperature).

Furthermore, the daily minimum values before sunrise during the 27 days were determined, as shown in Figure 13a. The air temperature values were generally lower than structural temperatures, with a difference ranging from 0.5 to 2.8 °C. Therefore, without the measured structural temperature data, the air temperature before sunrise can be adopted as the initial temperature value for transient thermal calculation [19]. In this study, the average values of the measuring points before sunrise were obtained as the initial value of the temperature field. Therefore, the accuracy of thermal numerical simulation could be improved.

#### 5.2.2. Equivalent Boundary Conditions

In the numerical analysis with ANSYS, the thermal load of solar radiation and radiation heat transfer can be applied on the FE model through the Neumann boundary conditions, but the convective heat transfer must be added through the Robin boundary conditions. Therefore, the Neumann boundary conditions are transformed into Robin boundary conditions for efficient computing by ANSYS, and the thermal boundary conditions defined as Equation (3) can be transferred into the equivalent boundary conditions for concrete bridges as per Equation (21):

(21) $$\lambda {\frac{\partial T}{\partial n}|}_{\Gamma }=h^{*}\left(T_{a}^{*}-T_{\Gamma }\right)$$

where $h^{*}$ is the coefficient of total heat transfer ($W\cdot m^{-2}\cdot K^{-1}$), and $h^{*}=h_{c}+h_{r}$; $T_{a}^{*}$ is the integrated atmospheric temperature, and $T_{a}^{*}=T_{a}+\left(q_{s}-q_{ra}\right)/h^{*}$.

## 6. Results and Discussions

### 6.1. Weather Conditions Records

According to observed historical data, the daily weather conditions for the whole experimental period are listed in Table 2. The weather condition can be categorized into sunny, cloudy, overcast, and rainy conditions, based on the cloud amount and rainfall level. There were seven days recorded in obviously overcast or rainy (O/R) conditions. The corresponding numerical simulation results of these seven days will be discussed separately in Section 6.2.

**Figure 14:**
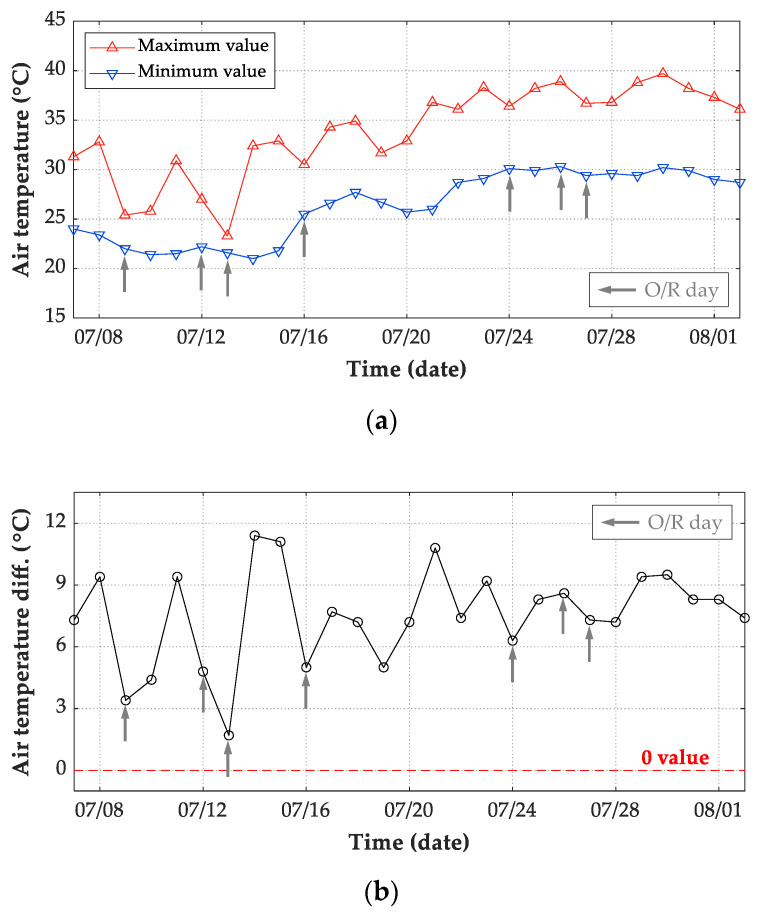
Daily air temperature records from 7 July 2019 to 2 August 2019. (**a**) Daily maximum and minimum value; (**b**) daily difference (maximum minus minimum value).

The meteorological records of air temperature, wind speed, and solar radiation were adopted as input in the FE model. For thermal boundary conditions of transient analysis, both convection and radiation heat transfer flux are determined by air temperatures. Therefore, the presentation of measured air temperatures is essential to understand the thermal behavior of concrete structures. For the period extended from 7 July 2019 to 2 August 2019, Figure 14a shows the daily maximum and minimum air temperatures. The maximum air temperature of the 27 days was 39.7 °C, which was recorded on 30 July 2019 with cloudy condition, while the minimum air temperature was 21.0 °C recorded on 14 July 2019 with sunny condition. The difference between the daily maximum and minimum temperatures is also required to understand the structural temperatures. During this period, the maximum air temperature difference was recorded on 14 July 2019 and was 11.4 °C, as shown in Figure 14b.

**Figure 15:**
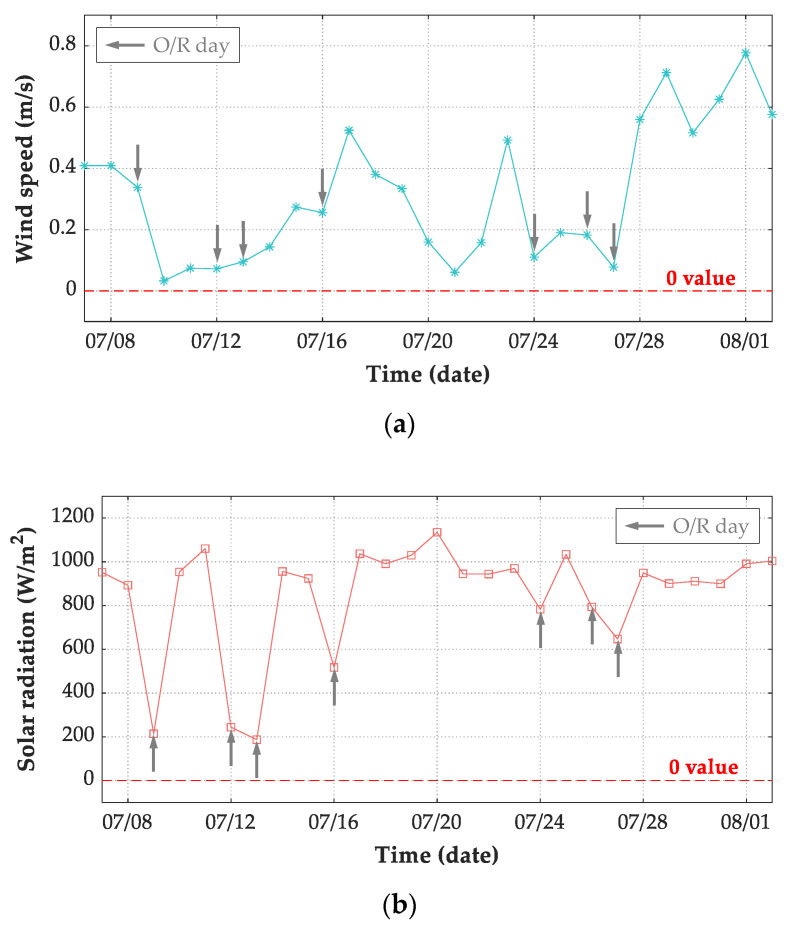
Daily environmental records from 7 July 2019 to 2 August 2019. (**a**) Average wind speed; (**b**) maximum solar radiation every 10 minutes.

As wind speed is also an influential factor on convection heat transfer flux, the daily average wind speed for the whole record period is presented in Figure 15a. It is obvious that the daily average wind speed ranged from 0 m/s to approximately 0.8 m/s. Meanwhile, during the test period, the recorded maximum wind speed was 2.2 m/s, while a daily minimum wind speed of 0 m/s was frequent. Both the air and structural temperatures are dominated by solar radiation during the appearances of sunrays. The air is warmed by sunrays under sunny conditions during daytime and cooled without sunrays during night hours. Therefore, solar radiation has high intensity during daytime on S/C days and low values on O/R days. The daily maximum solar radiation for the test period is plotted in Figure 15b. The maximum recorded value of the daily maximum hourly global solar radiation was recorded on 20 July 2019 with sunny condition, which was 1135 $W/m^{2}$. Meanwhile, daily maximum solar radiations less than 300 $W/m^{2}$ were recorded in heavily overcast or rainy days, such as 09 July 2019, 12 July 2019, and 13 July 2019. Therefore, daily maximum solar radiations intensity on O/R days were generally lower than that on S/C days.

### 6.2. Evaluation of FE Simulation Results Based on Measured Records

**Figure 16:**
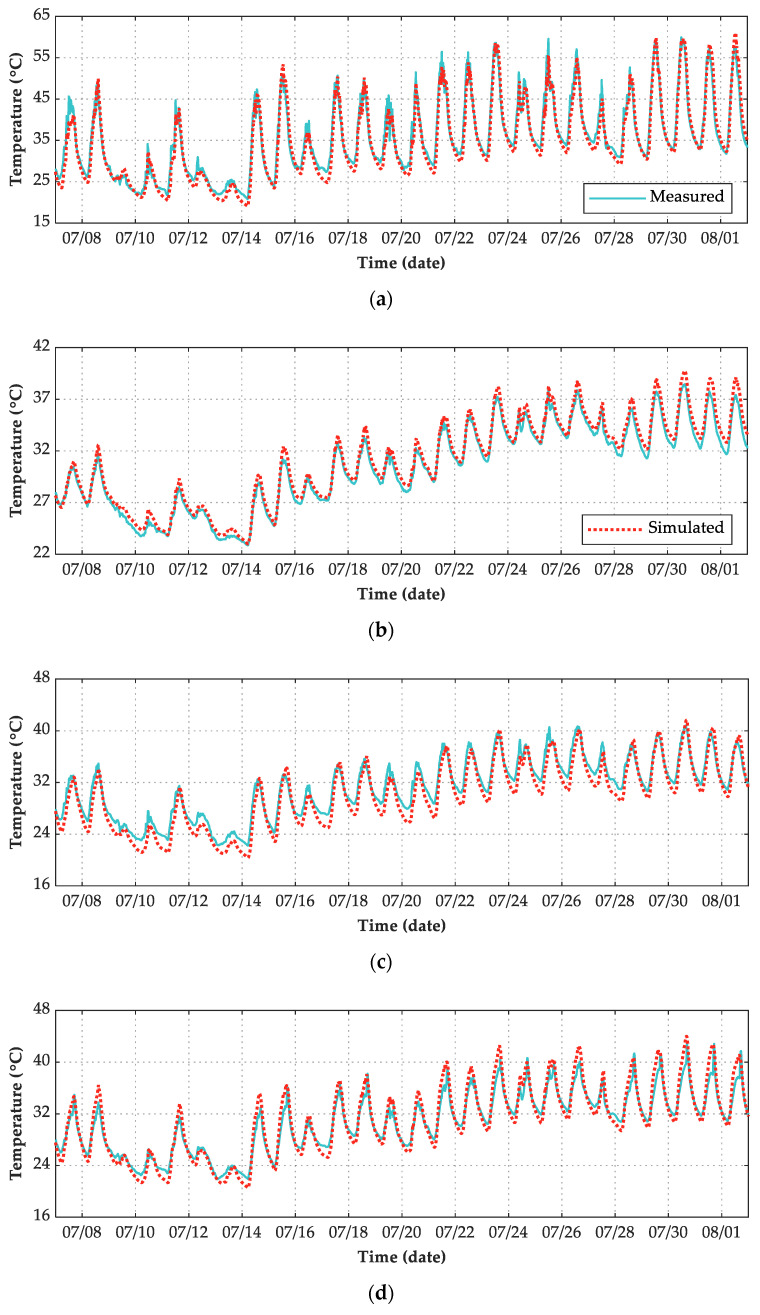

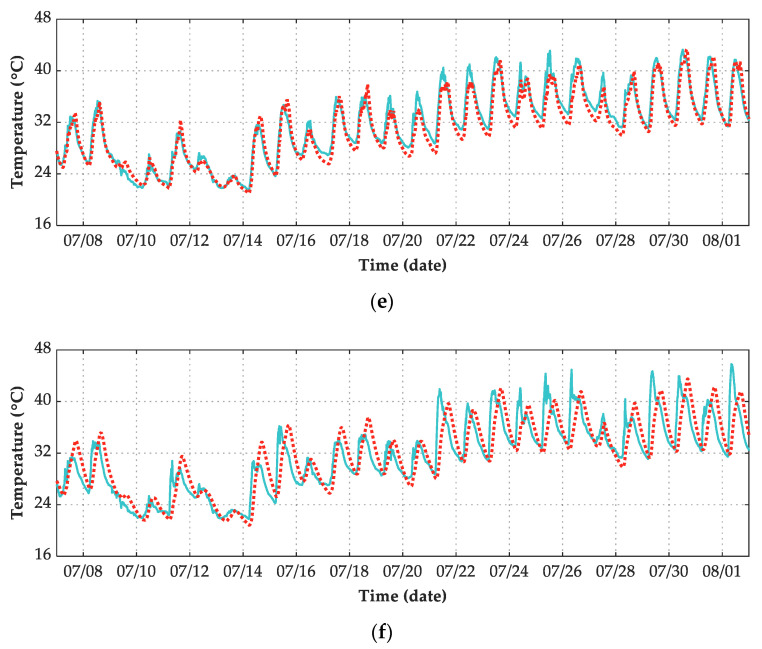
Time-temperature curve of 6 points. (**a**) TS; (**b**) BS; (**c**) SS; (**d**) NS; (**e**) SP; (**f**) EP.

Figure 16 shows the comparison between numerical simulation temperature results and measured temperature results of the maglev bridge from 7 July 2019 to 2 August 2019. The time-temperature curve illustrates that except for the EP point on the east surface of the pier, through establishing a reasonable FE model and adopting proper boundary conditions, the ANSYS simulation results coincided well with the experimental results.

Table 2 between simulated value and the measured value is used to evaluate the numerical simulation results, as expressed as Equation (22):

(22) $$R^{2}=\frac{\sum_{i=1}^{N}\left(X_{Si}-{\bar{X}}_{S}\right)\left(X_{Mi}-{\bar{X}}_{M}\right)}{\sqrt{\sum_{i=1}^{N}{\left(X_{Si}-{\bar{X}}_{S}\right)}^{2}\sum_{i=1}^{N}{\left(X_{Mi}-{\bar{X}}_{M}\right)}^{2}}}$$

where $X_{Si}$ is the numerical simulation result, $X_{Mi}$ is the corresponding experimental record at the same time step, and N is the number of calculation steps or records; ${\bar{X}}_{S}$ is the average simulation calculation value; and ${\bar{X}}_{M}$ is the average experimental measured value. When $0.95\leq \left|r_{SM}\right|\leq 1$, the simulation results coincide well with the experimental results; otherwise, the simulation results are not good. Meanwhile, two error indexes are used in addition to the correlation coefficient, they are average absolute error (AAE) and root-mean-square error (RMSE), as expressed as Equations (23) and (24), respectively.

(23) $$AAE=\frac{\sum_{i=1}^{N}\left|X_{Si}-X_{Mi}\right|}{N}$$

(24) $$RMSE=\sqrt{\frac{\sum_{i=1}^{N}{\left(X_{Si}-X_{Mi}\right)}^{2}}{N}}$$

According to the daily weather records listed in Table 2, statistics of three evaluation indexes in three kinds of weather conditions are listed in Table 3. In terms of the $R^{2}$ index, the numerical simulation results in O/R days are better than those in S/C days. For the measuring point on the east surface of the pier (EP), the regression coefficient index is lower than 0.90 in all-weather conditions and S/C days, so the simulation results are not good. The simulation results of the rest measuring points coincided well with the experimental results. In terms of AAE index and RMSE index, except for the measuring point on the south surface of the beam (SS), the simulation results errors of other measuring points are lower in O/R days than those in S/C days. The errors of measuring point EP are greater than that of other measuring points in all-weather and S/C conditions.

Intuitive plots of simulated results and measured records for six measuring points are shown in Figure 10, in which each dot corresponds to a calculation and measurement step. In these figures, the further away the dot is from the baseline, the larger the error is. Here, the maximum error rate (ME) illustrates the deviation level as Equation (25):

(25) $$ME=\{\begin{array}{cc} \max\left(X_{Si}-X_{Mi}/X_{Mi}\times 100\%\right) & X_{Si}>X_{Mi},\\ \min\left(X_{Si}-X_{Mi}/X_{Mi}\times 100\%\right) & X_{Si}<X_{Mi} \end{array},\;i=1,2,\cdots ,N$$

**Figure 17:**
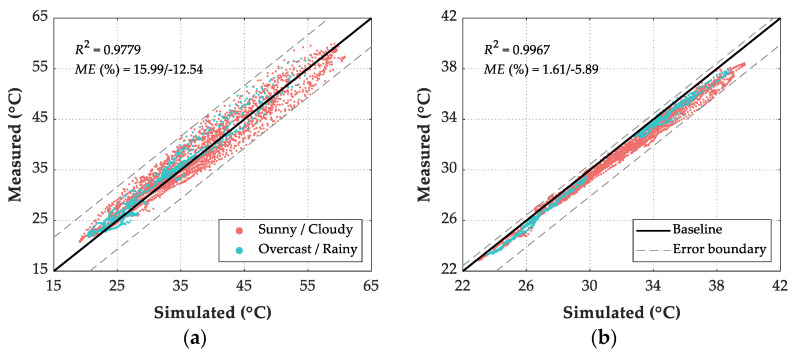

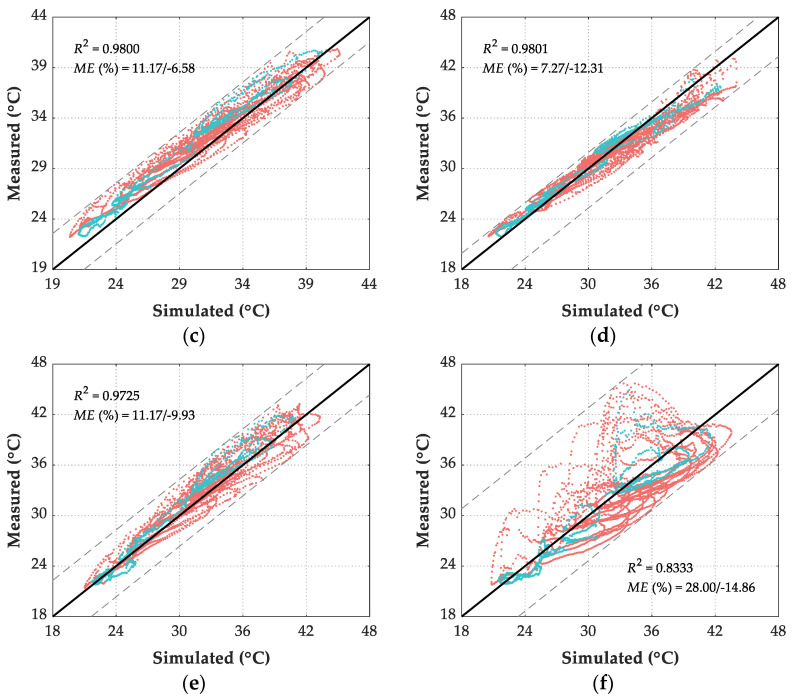
Comparison between FE simulated results and measured records of 6 points. (**a**) TS; (**b**) BS; (**c**) SS; (**d**) NS; (**e**) SP; (**f**) EP.

Figure 17b shows that the simulated results of measuring point BB coincided best with their corresponding experimental values with $R^{2}$ of 0.9967, while the ME were 1.61% and −5.89%. Figure 17f shows that the simulated results of measuring point EP coincided worst with their corresponding experimental values with $R^{2}$ of 0.8333, while the ME were 28.00% and −14.86%. The simulated results of the rest measuring points, including TS, SS, NS, and SP, coincided well with their experimental values with $R^{2}>0.95$, while the ME ranged from −12.54% to 15.99%. Overall, the proposed numerical simulation method of structural temperature field can be suitable for the concrete bridge structure under various weather conditions.

### 6.3. Temperature Distributions in Sunny and Rainy Conditions

**Figure 18:**
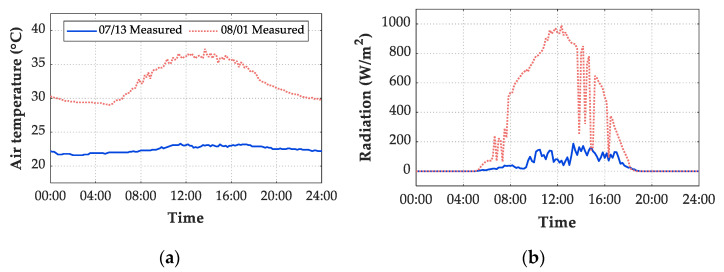
Time variation of meteorological data during one day. (**a**) Air temperature; (**b**) total solar radiation on the ground.

Figure 18 shows the time history of air temperature and total solar radiation on the ground on two selected days. The amount of radiation on 13 July 2019 in the rainy condition was much smaller than that on 1 August 2019 in the sunny condition. Due to the influence of solar radiation, the daily structural temperature difference values on rainy days were relatively small, whereas the difference values on sunny days were high.

**Figure 19:**
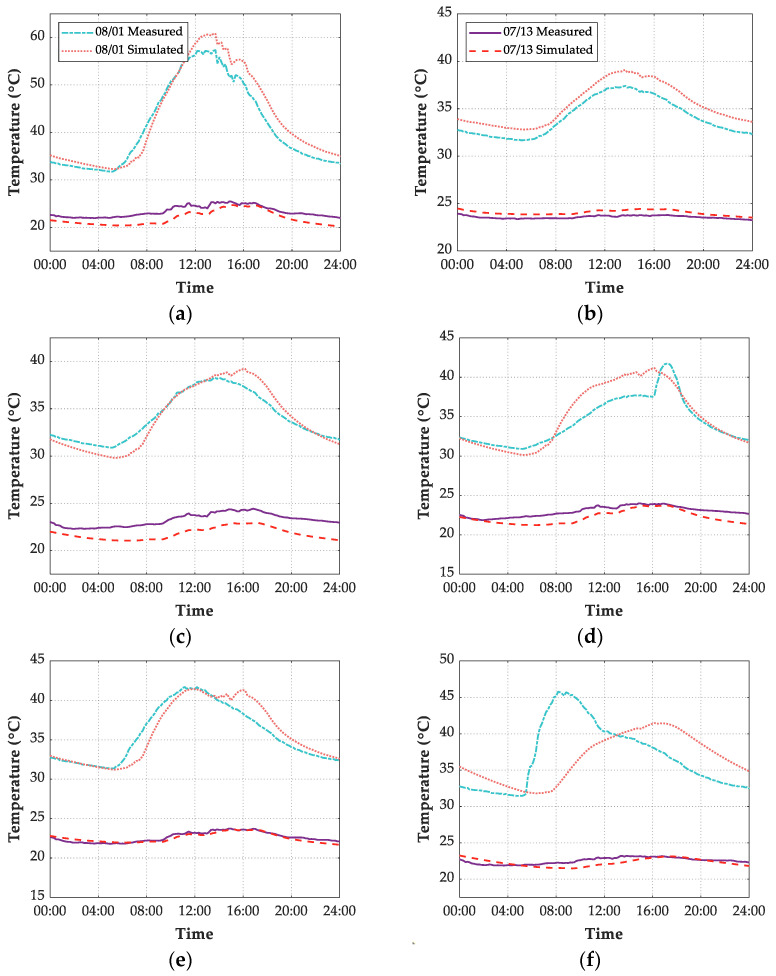
Comparison between FE simulated results and measured records of 6 points on 13 July 2019 and 1 August 2019. (**a**) TS; (**b**) BS; (**c**) SS; (**d**) NS; (**e**) SP; (**f**) EP.

According to weather conditions analyzed in Section 6.1, two days under different weather conditions were selected to visualize the daily simulated and measured gradients, as Figure 19. These days are 1 August 2019 and 13 July 2019, with sunny and rainy weather conditions, respectively. Figure 19 shows that the simulation results of six measuring points coincided well with measured records in the rainy weather. Except for the measuring point EP on the east surface of the pier, the simulation results of the rest five measuring points were consistent well with the measured values, and the maximum error is acceptable with approximately 4 °C.

Because of the high solar altitude angles in summer, solar radiation was concentrated on the top structural surface during the mid-day hot hours on a sunny day, leading to the highest simulated and measured temperature on the top, as shown in Figure 19a. The sunray with high altitude angles also caused the most area of the south surface on the beam in the solar shadow. Therefore, the simulated and measured temperature values of the SS point and NS point on the beam surface were relatively close, as shown in Figure 19c,d.

As shown in Figure 19f, the simulated temperature peaks of EP point occurred later than that of measured temperature peaks on sunny days. It is necessary to highlight the reason behind the large error between simulated results and measured records of measuring point EP. On the one hand, the cosine response of the pyranometer (BL-ZFS) is ≤±5%, which means the measured solar radiation records were inaccurate at sunrise or sunset when solar altitude angle is $0{}^{\circ}\leq \beta_{s}\leq 10{}^{\circ}$. Therefore, a cosine corrector installed on a pyranometer compensates for the measurement inaccuracy at sunrise or sunset in the future. On the other hand, the empirical formula of diffuse sky radiation and atmospheric cleanliness index, shown as Equation (12), may not be applicable at sunrise and sunset conditions, which causes the calculated solar radiation inaccurate. Overall, installing the direct solar pyranometer is recommended to track the sunray in real-time and measure real-time direct solar radiation, solar azimuth, and solar altitude angle, thus improving the temperature simulation accuracy of the east or west surface on the structure.

## 7. Conclusions

With a special focus on accurate simulation of the structural temperature field, an all-weather numerical simulation methodology considering complicated thermal boundary conditions was presented to predict the temperature distribution of concrete bridge structures. The proposed method was performed on a concrete maglev bridge based on real-time 27-day meteorological data in summer. An experimental program on a maglev bridge was conducted to demonstrate the proposed methodology to discuss the reliability and accuracy under various weather conditions in summer. Based on the results, the following conclusions are drawn as follows:

(1) Based on real-time measured meteorological data and the empirical formula, the complicated thermal boundary conditions of the bridge could be calculated to realize the accurate bridge temperature field simulation. Mean values of structural temperature measured by sensors before sunrise can be adopted as initial value conditions for transient thermal analysis.
(2) Solar radiation dominates the thermal energy exchange between structures and the environment during the daytime. For the concrete bridge structures under solar radiation, the proposed simulation algorithm of solar shadow and the reasonable radiation flux model provide the optimum compromise of accuracy, which is the basis of accurate numerical simulation.
(3) The proposed temperature field model is more suitable for cloudy or rainy days. The comparison of simulated and measured results in cloudy or rainy days and those in sunny or cloudy days shows that the accurate measurement of direct solar radiation from sunrise to sunset plays a significant role in the accuracy of temperature field simulation.
(4) Because of the high solar altitude angles in summer, solar radiation was concentrated on the structural top surface of the beam, leading to the highest temperature. Besides, the north and south surfaces of the beam were shielded by the top plate most of the time, which made the small temperature difference between the two sides.

Further work will adopt the presented methodology to simulate the structural temperature in winter with low solar altitude angles. A more detailed investigation of the stress caused by thermal loads will be carried out.

## Figures and Tables

**Table 1:** Parameters and values adopted in FE model.

Concrete Material Properties			Geography Information		
Density ρ	Specific Heat Capacity c	Thermal Conductivity λ	Latitude φ	Longitude α	$\mathrm{Southward}\;\mathrm{Azimuth}\;\alpha_{n0}$
$\left(\mathrm{kg}\cdot m^{-3}\right)$	$\left(J\cdot {\mathrm{kg}}^{-1}\cdot K^{-1}\right)$	$\left(W\cdot m^{-1}\cdot K^{-1}\right)$	(°)	(°)	(°)
2500	1114	2.326	31.297 N	121.217 E	0

**Table 2:** Daily weather records from 7 July 2019 to 2 August 2019.

**Order**	**1**	**2**	**3**	**4**	**5**	**6**	**7**	**8**	**9**
**Date**	07 July	8 July	9 July	10 July	11 July	12 July	13 July	14 July	15 July
**O/R**			√			√	√		
**Weather**	C	C→R	R+→R	R→C	C→O	R→R++	R→C	C	C
**Order**	**10**	**11**	**12**	**13**	**14**	**15**	**16**	**17**	**18**
**Date**	16 July	17 July	18 July	19 July	20 July	21 July	22 July	23 July	24 July
**O/R**	√								√
**Weather**	R	R→C	C	C	C→S	C	C	C	O
**Order**	**19**	**20**	**21**	**22**	**23**	**24**	**25**	**26**	**27**
**Date**	25 July	26 July	27 July	28 July	29 July	30 July	31 July	1 August	2 August
**O/R**		√	√						
**Weather**	C	R→O	R	C→S	C	O→C	C	S	S

S: sunny; C: cloudy; O: overcast; R: lightly rainy; R+: moderately rainy; R++: heavily rainy; →: weather change.

**Table 3:** Calculated results of evaluation index.

Index	Weather Condition	Measuring Point					
		TS	BS	SS	NS	SP	EP
$R^{2}$	All	0.9779	0.9967	0.9800	0.9801	0.9725	0.8333
	S/C	0.9752	0.9959	0.9757	0.9763	0.9656	0.7733
	O/R	0.9856	0.9990	0.9924	0.9891	0.9898	0.9430
AAE	All	1.687	0.605	1.339	1.074	1.109	2.301
	S/C	1.771	0.647	1.306	1.155	1.138	2.637
	O/R	1.446	0.488	1.433	0.842	1.025	1.341
RMSE	All	2.104	0.710	1.529	1.337	1.393	3.143
	S/C	2.206	0.764	1.519	1.432	1.422	3.455
	O/R	1.781	0.528	1.559	1.019	1.308	1.998

## Data Availability

Data is contained within the article.
